# A Novel Medical Image Protection Scheme Using a 3-Dimensional Chaotic System

**DOI:** 10.1371/journal.pone.0115773

**Published:** 2014-12-26

**Authors:** Chong Fu, Gao-yuan Zhang, Ou Bian, Wei-min Lei, Hong-feng Ma

**Affiliations:** 1 School of Information Science and Engineering, Northeastern University, Shenyang, China; 2 The General Hospital of Shenyang Military Command, Shenyang, China; 3 TeraRecon, Foster City, California, United States of America; University of Catania, Italy

## Abstract

Recently, great concerns have been raised regarding the issue of medical image protection due to the increasing demand for telemedicine services, especially the teleradiology service. To meet this challenge, a novel chaos-based approach is suggested in this paper. To address the security and efficiency problems encountered by many existing permutation-diffusion type image ciphers, the new scheme utilizes a single 3D chaotic system, Chen's chaotic system, for both permutation and diffusion. In the permutation stage, we introduce a novel shuffling mechanism, which shuffles each pixel in the plain image by swapping it with another pixel chosen by two of the three state variables of Chen's chaotic system. The remaining variable is used for quantification of pseudorandom keystream for diffusion. Moreover, the selection of state variables is controlled by plain pixel, which enhances the security against known/chosen-plaintext attack. Thorough experimental tests are carried out and the results indicate that the proposed scheme provides an effective and efficient way for real-time secure medical image transmission over public networks.

## Introduction

### A. Background

Telemedicine or telehealth, a product of 20th century telecommunication and information technologies, is emerging as a critical component of the healthcare crisis solution. It holds the promise to significantly impact some of the most challenging problems of our current healthcare system: access to care, cost effective delivery, and distribution of limited providers. As is known, medical applications often deal with patients' data that are confidential, and it must be ensured that medical data are collected and communicated securely, accessed by authorized persons only. This is even crucial for telemedicine/telehealth services as they inevitably involve the transmission of medical, imaging and health informatics data over open networks such as the Internet. Nowadays, preserving the privacy of medical data is not only an ethical but also a legal requirement [1]–[4]. For instance, the Health Insurance Portability and Accountability Act (HIPAA) [5], enacted by the United States Congress and signed by President Bill Clinton in 1996, obliges health care institutions to take proper measures to ensure that patients' information is only accessible to people who have a professional need. Moreover, several major medical imaging communities such as American College of Radiology (ACR) and Society of Computer Applications in Radiology (SCAR) have issued guidelines and mandates for ensuring medical image security.

A direct and obvious way to protect medical data from unauthorized eavesdropping is to use an encryption algorithm. However, conventional block ciphers, such as Triple-DES, AES and IDEA, are not suitable for practical medical image cipher due to the size of image data and increasing demand for real-time teleradiology and other online telehealth services. To meet this challenge, many different encryption technologies have been proposed. Among them, chaos-based algorithms have suggested a promising direction [6]–[33]. Making use of the favorable characteristics such as high sensitivity to initial condition and parameters, ergodicity and pseudo-randomness, chaotic systems have demonstrated great potential for information especially multimedia encryption. In 1998, Fridrich [6] proposed the first chaos-based image encryption scheme, which consists of two major steps: permutation and diffusion. In the first step, almost all the pixels are rearranged in a pseudorandom manner, which leads to a great reduction in the correlation among adjacent pixels. In the second step, the pixel values are altered sequentially and the modification made to a particular pixel usually depends on the accumulated effect of all the previous pixel values. As a result, a minor change in one pixel of the plain image may result in a totally different cipher image with several overall rounds of encryption. The architecture of the proposed scheme formed the basic structure for many of the chaos-based image encryption techniques that are presented later in the literature.

### B. Related work

Following Fridrich's pioneer work, a number of chaos-based image cryptosystems utilizing different chaotic systems, their improvements, and cryptanalysis have been proposed [7]–[36]. Among these schemes, the permutation operations are almost exclusively realized by three types of area-preserving invertible chaotic maps, i.e., Arnold cat map [8], [22], [28]–[29], [31], [33], baker map [6]–[7], [9], [19], and Chirikov standard map [10], [17], [20], [22], [27], [30]. Their discretized versions are given by Eqs. (1) – (3), respectively.
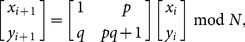
(1)

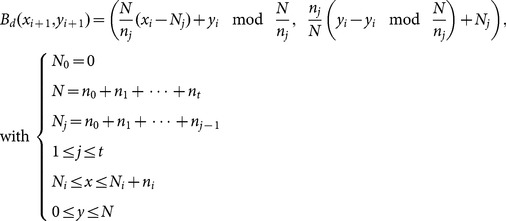
(2)

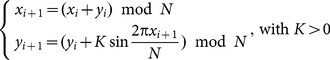
(3)where *N* is the width or length of a square image, (*x*, *y*) is the pixel position in the image, mod(*x*, *y*) divides *x* by *y* and returns the remainder of the division, and *p*, *q*, *n_i_* and *K* are parameters that control the permutation and accordingly serve as part of the secret key, i.e., the permutation key.

This kind of permutation strategy, benefit from the desirable properties of a nonlinear dynamical system, suffer from two main disadvantages: (1) As is known, an aperiodic chaotic map may become periodic after discretization [8]. That is, image randomized by the transformation may return to its original state after a number of iterations. (2) As these descretized maps are defined on a finite square lattice of points which represent pixels, an extra transformation is required when dealing with a general rectangular image. To address above drawbacks, several improved approaches for image permutation has been developed. For instance, Gao et al. [14] proposed an image permutation algorithm which utilizes a total shuffling matrix derived from chaotic logistic map. This method can produce a satisfactory scrambling effect, but suffers from unsatisfied time consumption. This is because a heavy computational load is involved in producing a sequence of unique pseudorandom positions. In [29], Fu et al. proposed an efficient permutation scheme using a chaotic sequence sorting algorithm. Unfortunately, the effectiveness of this method is not so good as the basic permutation unit is a whole row/column of the image rather than a pixel or even a bit.

In the diffusion stage, one-dimensional (1D) chaotic maps, such as logistic map, skew tent map and Chebyshev map, are widely employed to generate pseudorandom keystream owing to the advantage of simplicity and high efficiency [8]–[11], [17], [20], [22], [28], [30]–[31], [33]. However, the weaknesses of these low-dimensional chaotic system based schemes, such as small key space and weak security, are also obvious. To address this issue, many researchers turn to find some improved chaos-based cryptosystems with large key space and good diffusion mechanisms. For instance, Behnia et al. [15] suggest a way of improving the security of chaos-based cryptosystem by using hierarchy of one dimensional chaotic maps and their coupling, which can be viewed as a high dimensional dynamical system. Gao et al. [16] reported an image encryption algorithm based on hyperchaos, whose states combinations are used to change the grey values of the shuffled image. Compared with ordinary chaotic systems, hyperchaotic systems, possessing more than one positive Lyapunov exponents, have more complex dynamical behaviors and number of system variables, which ensure the strong unpredictability and large key space of a cryptosystem. Sun et al. [18] presented an approach using spatial chaos system for high degree security image encryption. The basic idea is to encrypt the image in space with spatial chaos map pixel by pixel, and then the pixels are confused in multiple directions of space. Rhouma et al. [21] proposed an OCML-based color image encryption scheme with a stream cipher structure. In this scheme, an external key of 192-bit length is chosen to generate the initial conditions and the parameters of the OCML by making some algebraic transformations so as to enhance the sensitivity to the change of any bit of the key. Amin et al. [25] introduced a new chaotic block cipher algorithm for image cryptosystems. By using 256-bits session keys, this scheme encrypts 256-bits input plain image to 256-bits output cipher image based on chaotic tent map. Seyedzadeh et al. [32] presented a chaos-based image encryption algorithm by using a Coupled Two-dimensional Piecewise Nonlinear Chaotic Map (CTPNCM), whose initial conditions and parameters are generated from a 256-bit long secret key.

Despite above notable achievements made in recent years, many existing chaotic image cryptosystems still suffer from some common cryptographic attacks, especially the known/chosen-plaintext attack [34]–[36]. This is because the diffusion keystream used in most schemes is solely determined by the key, whereas none of these cryptosystems employ a one-time pad mechanism. That is, the same keystream is used to encrypt different plain images unless a different key is used. The keystream can be easily determined by encrypting some special images (e.g. an all-white or all-black image) and then comparing them with the corresponding cipher images. To address this problem, Wang et al. [22] proposed a plain image related keystream generation scheme. In their scheme, the keystream elements are extracted from multiple times iteration of a chaotic map, and the iteration times is determined by plain pixel values. However, as the iterations of a chaotic map have to involve the real number arithmetic operation, the extra iteration operations degrade the performance of the cryptosystem to some extent.

Apart from security considerations, performance is another fundamental issue for an image cryptosystem. Recent studies have pointed out that the diffusion procedure is the highest cost, in term of computational times, of the whole cryptosystem [17], [23], [30]. This is because a considerable amount of computation load is needed to deal with the real number arithmetic operation and the subsequent quantization required by the keystream generation. Consequently, approaches on performance improvements are mainly focus on how to effectively reduce the number of diffusion (overall) rounds or the computational complexity of diffusion operation without downgrading the security level. For instance, Xiang et al. [12] proposed a selective image encryption method that only encrypts the four higher bits of each pixel by the keystream generated from a one-way coupled map lattice. This algorithm has a reduced execution time as it only encrypts 50% of the whole image data. Wong et al. [17] suggested to introduce certain diffusion effect in the permutation stage by simple sequential add-and-shift operations. The purpose is to reduce the workload of the time-consuming diffusion part so that fewer overall rounds and hence a shorter encryption time is needed. In [28], [31], [33], bit-level permutation algorithms were suggested for the same purpose. Wong et al. [23] proposed an efficient diffusion mechanism using simple table lookup and swapping techniques as a light-weight replacement of the 1D chaotic map iteration. Wang et al. [26] proposed a fast image encryption algorithm with combined permutation and diffusion. In their scheme, the image is firstly partitioned into blocks of pixels, and then, spatiotemporal chaos is employed to shuffle the blocks and, at the same time, to change the pixel values. Fu et al. [30] proposed a fast image cipher using a novel bidirectional diffusion. Simulation results indicated that their scheme requires only one round permutation and two rounds diffusion to achieve a satisfactory level of security.

### C. Our proposal

In this paper, we suggest a novel chaos-based image cipher for medical image protection. The new scheme utilizes a single 3D chaotic system, Chen's chaotic system, for both permutation and diffusion. In the permutation stage, we introduce a novel shuffling mechanism, which shuffles each pixel in the plain image by swapping it with another pixel at a location chosen by two of the three state variables of Chen's chaotic system. The remaining variable is used for quantification of pseudorandom keystream for diffusion. Compared with the permutation methods based on area-preserving chaotic maps, the new method avoids the drawback of short periodicity of permutation and can be directly applied to non-square images. Moreover, the selection of state variables is controlled by the plain pixel. As a result, the quantified keystream is related not only to the key but also to the plain image, which enhances the security against known/chosen-plaintext attack. The results of running speed test show the new scheme has a superior performance compared with some typical block and chaos-based approaches. The remainder of this paper is organized as follows. Section 2 discusses the new image encryption algorithm using Chen's chaotic system and how it is integrated into a teleradiology system. In Section 3, the effectiveness and efficiency of the proposed permutation method is analyzed and compared with those of existing methods. In Section 4, we analyze the security of the proposed image cipher and evaluate its performance through key space analysis, statistical analyses, key sensitivity analysis, differential analysis and speed analysis. Finally, conclusions are drawn in the last section.

## Medical Image Protection Using Chen's Chaotic System

### A. The Chen's chaotic system

In 1963, Edward Lorenz, an early pioneer of chaos theory, developed a simplified mathematical model for atmospheric convection. The model is a system of three ordinary differential equations now known as the Lorenz equations. Following his approach, Chen and Ueta constructed another 3D autonomous chaotic system with the method derived from engineering feedback control [37]. Though the two chaotic systems have a similar structure, they are not topologically equivalent and the Chen's system shows even more complex dynamical behaviors. The so-called general parametric Chen system is described by
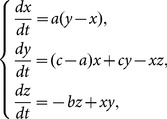
(4)where *a*, *b* and *c* are real parameters. The system is chaotic on a small subset {*a*, *b*, *c*}  =  {35, 3, 28} inside the 3D real parameter space, but for other parameter sets it may not be chaotic. For further details about the chaotic dynamics of the Chen's system, interested readers can refer to [38]. Obviously, the initial state values (*x*_0_, *y*_0_, *z*_0_), which uniquely determine the chaotic orbit and the consequent quantified keystream, can quite properly serve as the diffusion key.

### B. Encryption algorithm

Without loss of generality, we assume the plain image is of *W*×*H* pixels. The detailed encryption process is described as follows:

***Step 1***: Pre-iterate Eq. (4) for *N*_0_ times to avoid the harmful effect of transitional procedure, where *N*_0_ is a constant. To solve the equation, fourth-order Runge-Kutta method is employed, as given by
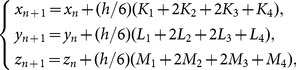
(5)where



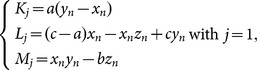


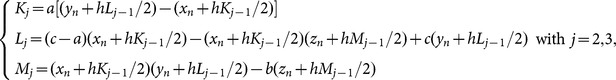


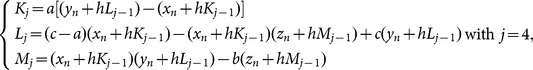
and *h* is the step size, which should be appropriately selected. Generally, the smaller the step size the more accurate the approximation. However, too small a step size will not help to yield better approximations as it will have roundoff error on a computer or calculator. When the roundoff error overwhelms the “discretization error”, the approximation will get bad and hence downgrade the randomness properties of its quantified keystream, especially the cross-correlation property. Therefore, *h* should be picked small enough that the answer is sufficiently accurate but not so small that roundoff error builds up too great. In our scheme, *h* is chosen as an empirical value of 0.0005.

***Step 2***: The Chen's system is iterated continuously. For each iteration, we can get three state values and one is selected as quantification of diffusion keystream according to
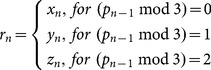
(6)where *p_n_*_-1_ is the previously operated plain pixel. One may set initial value *p*_0_ as a constant.

***Step 3***: The keystream element is quantified by using the following formula



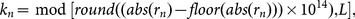
(7)where *abs*(*x*) returns the absolute value of *x*, *floor*(*x*) returns the value of *x* to the nearest integers less than or equal to *x*, *round*(*x*) rounds *x* to the nearest integers, and *L* is the color level (for a 256 grey-scale image, *L* = 256). In our scheme, all the state variables are declared as 64-bit double-precision type. According to the IEEE floating-point standard [39], the computational precision of the 64-bit double-precision number is about 10^−15^. Therefore, the fractional part of a state variable is multiplied by 10^14^ so as to ensure both the randomness and accuracy of the quantified keystream.

***Step 4***: Buffer keystream element *k_n_* into a vector *k* = {*k*_1_, *k*_2_, …, *k_W×H_*} as the diffusion operation is performed after permutation operation.

***Step 5***: Let *s_n_* and *t_n_* denote the remaining two state variables of the Chen's system. Swap current pixel with the pixel at position (*m*, *n*), where
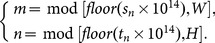
(8)


***Step 6***: Return to ***Step 1*** until all the pixels in the plain image are swapped from left to right, top to bottom.

***Step 7***: Modify the pixel values sequentially from left to right, top to bottom, during which the influence of each individual pixel is spread out over all its subsequent pixels in the image. This is done by using Eq. (9).
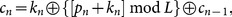
(9)where *p_n_*, *c_n_* and *c_n_*_-1_ are the currently operated pixel, output cipher pixel and previous ciphered pixel, respectively, and ⊕ performs bit-wise exclusive OR operation. Similarly, the initial value *c*_0_ may be set as a constant.

In general, 3–4 rounds of such permutation-diffusion operations are needed to achieve a satisfactory level of security. To accelerate the diffusion process, the shuffled image is diffused in order from bottom to top, right to left in every other round. With such a mechanism, the proposed scheme requires only two encryption rounds to achieve a satisfactory level of security.

### C. Decryption algorithm

In general, the decryption procedure is similar to that of the encryption process except that some steps are followed in a reversed order. However, there are still some slight differences between the two processes as the permutation table and the diffusion keystream are generated from Chen's system simultaneously. Moreover, as the proposed cryptosystem is a symmetric key cipher, the same secret key (*x*_0_, *y*_0_, *z*_0_) and initial conditions (*p*_0_, *c*_0_) should be used for decryption. The detailed decryption process is described as follows:

***Steps 1*** to ***3*** are the same as those of the encryption algorithm, except *p_n_*_-1_ denotes the previously deciphered pixel.

***Step 4***: Buffer (*s_n_*, *t_n_*) into a *W*-by-*H*-by-2 permutation matrix *M_p_* as the decryption is done in reverse order of encryption.

***Step 5***: Remove the effect of diffusion from the cipher image to obtain an intermediate image, i.e., the shuffled image. The detailed operations are the same as those described in ***Step 7*** in encryption, except that the inverse of Eq. (9) is applied, as given by



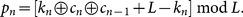
(10)


***Step 6***: Remove the effect of permutation from the shuffled image to recover the plain image. This is done by swapping the pixels of the shuffled image according to the permutation matrix *M_p_* in reverse order of ***Step 6*** in encryption, i.e., from, bottom to top, right to left. Obviously, matrix *M_p_* should also be used reversely.

As both decipher and encipher procedures have similar structures, they have essentially the same algorithmic complexity and time consumption.

### D. Integration of the proposed cryptosystem

**Figure 1:**
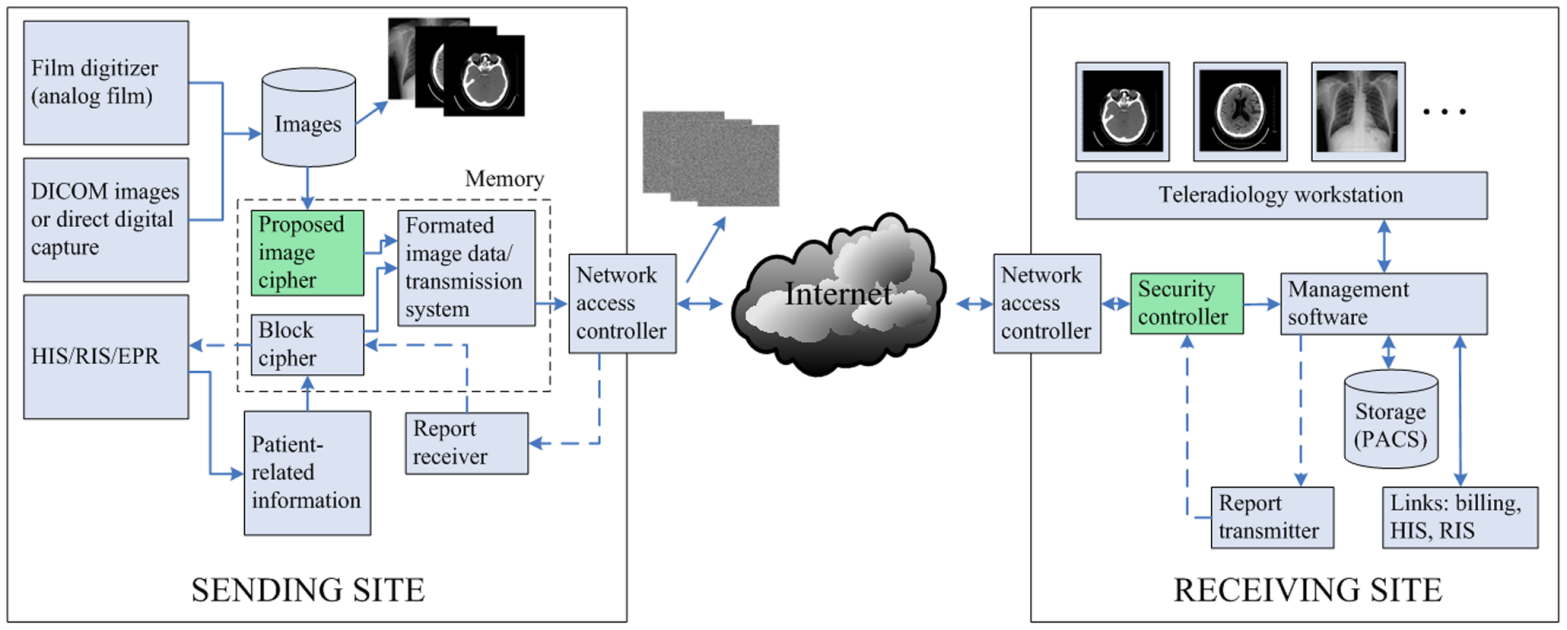
The integration of our proposed cryptosystem into a teleradiology system.

Our proposed cryptosystem can be easily integrated into a teleradiology system as an independent security module, as illustrated by Fig. 1.

As is known, medical images acquired from digital modalities (CT, CR, DR, MRI, DSA) are stored in a uniform DICOM (Digital Imaging and Communications in Medicine) format with a “.dcm” file extension [40]. According to DICOM standard, a DICOM file is in fact a combination of a pair of files, namely the header file (“.hdr”) and the image file (“.img”). The former contains the patient's as well as the hospital's data, commonly stored in an ASCII format, can be easily handled by a typical block cipher such as AES and Triple-DES, while the latter contains the pure image data which will be protected by the proposed cryptosystem. The image data in a DICOM file are usually stored in an uncompressed or lossless compressed format to keep all original information intact. Consequently, a compressed image should be firstly uncompressed to a “raw” format and then encrypted using the proposed scheme. After that, the ciphered image file can be recompressed by using a proper method according to the transfer requirement. Finally, the header file and the processed image file are recombined and a ciphered DICOM file to be transmitted is obtained. When the ciphered images arrive at the receiving end, they are decrypted directly in the memory and then stored in the PACS server for authorized access.

It's worth noting that medical imaging data are frequently three-dimensional and four-dimensional datasets with highly correlated consecutive images [41]. Compression makes full use of the correlations. Such characters do not have any impact on the security as our cryptosystem is highly sensitive to the plaintext. That is, even if two consecutive images have one bit difference, their corresponding resultant images will be totally different. Detailed plaintext sensitivity analysis will be carried out in Sec. 4.4. However, as the correlation no longer exists after encryption, the compression ratio of the ciphered images will be lower than that of plain images. Moreover, by using pixel as basic processing unit, our proposed scheme can flexibly deal with medical images of different resolutions. In other words, our cryptosystem is resolution-independent.

It can be seen from above discussion that there is no technical barriers to integrating such a cryptosystem into an existing teleradology system. The proposed encryption/decryption algorithms are suggested to be encapsulated in a DLL that can be flexibly invoked by a third-party data sending/receiving program. Moreover, as the proposed scheme is fully software-implemented, no extra hardware and its associated cost are needed by either site.

## Permutation Performance Analysis

**Figure 2:**
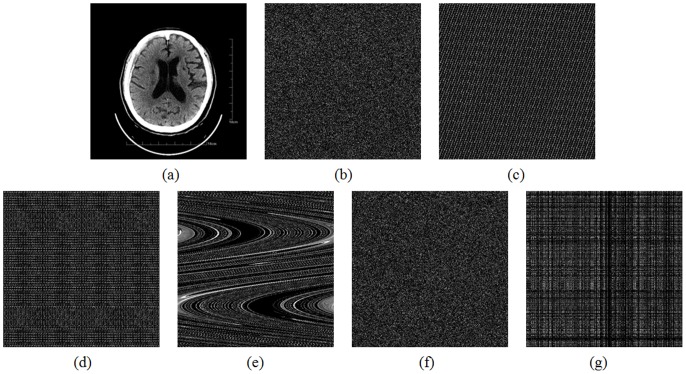
The application of the proposed and five comparable permutation methods.

Fig. 2 demonstrates the application of the proposed and five comparable permutation methods to a grayscale head CT image with 512×512 size. Fig. 2(a) shows the plain image, and Table 1 lists the parameters used in each method, including the number of permutation rounds and the permutation key. The total shuffling and chaotic sequence sorting algorithms are based on chaotic logistic and Chebyshev maps, respectively. As can be seen from Table 1, only one round of operation is adopted for the proposed, the total shuffling and the chaotic sequence sorting methods as their effect are not sensitive to the number of rounds performed. While for other three area-preserving map based methods, three rounds of operation are performed to ensure the pixels in the plain image are sufficiently shuffled.

**Table 1:** Parameters used in the proposed and comparable permutation methods.

Shuffled image	Method employed	Round(s)	Permutation key
Fig. 2(b)	Proposed method	1	*x*_0_ = 6.3, *y*_0_ = 3.5, *z*_0_ = 9.9
Fig. 2(c)	Cat map	3	*p* = 40, *q* = 8
Fig. 2(d)	Baker map	3	*n_i = _*_0, …, 9_ = {64, 32, 32, 64, 64, 64, 64, 32, 32, 64}
Fig. 2(e)	Standard map	3	*K = *768
Fig. 2(f)	Total shuffling	1	*µ = *4.0, *x*_0_ = 0.3
Fig. 2(g)	Chaotic sequence sorting	1	*k = *4.0, *x*_0_ = 0.7

It's clear from Fig. 2 that permutation effect of the proposed and total shuffling methods are significantly better than that of the other four methods. There are still some textures can be found in Figs. 2(c), (d), (e) and (g), whereas the pixels in (b) and (f) of Fig. 2 are arranged in a perfectly random way.

To further quantify the effectiveness of a permutation method, the analysis of correlations of adjacent pixels is carried out, as discussed in the following. First, randomly select 5000 pairs of adjacent pixels in horizontal, vertical and diagonal direction from the shuffled image, respectively. Then, calculate the correlation coefficient *r_x_*_,*y*_ of each pair by using the following three formulas:
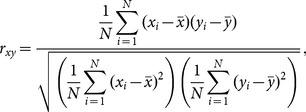
(11)

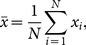
(12)

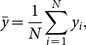
(13)where *x_i_* and *y_i_* are grayscale values of the *i*th pair of adjacent pixels, and *N* denotes the total number of samples.

**Figure 3:**
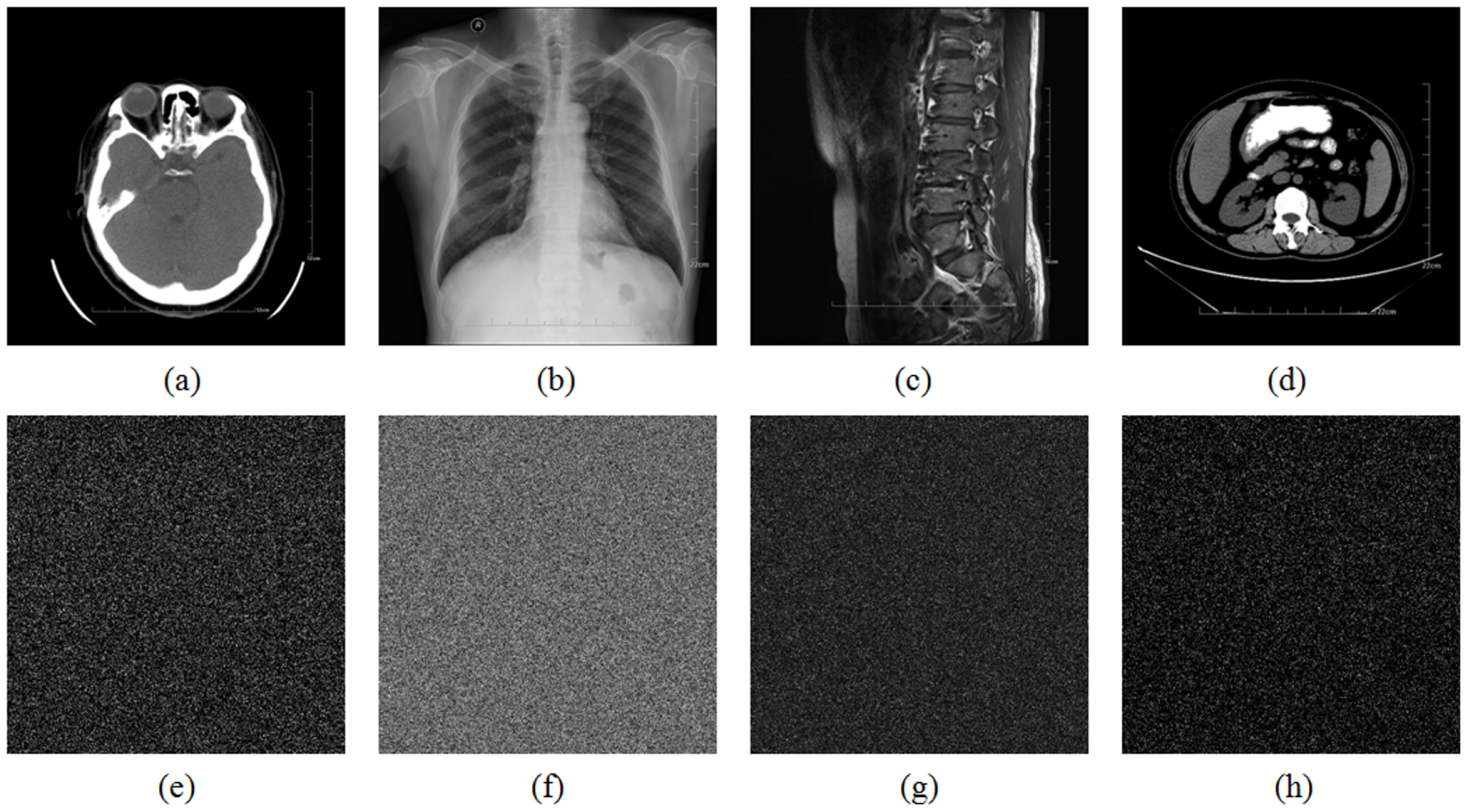
Test images and their corresponding shuffled images produced by the proposed method: (a) paranasal sinus CT. (b) chest X-ray. (c) waist MR. (d) abdomen CT. (e) – (h) are the shuffled images corresponding to (a) – (d), respectively.

To demonstrate the stability of our proposed method, four more medical images of different parts of the body are tested. Figs. 3(a) – (d) show the plain images and their corresponding shuffled images produced by the proposed method are shown in Figs. 3(e) – (h), respectively.

Table 2 lists the results of the correlation coefficients of adjacent pixels for different test images and their corresponding shuffled images produced by the proposed and the comparable methods. As the pixel pairs are randomly selected, the test is repeated 8 times for each direction and the mean value is calculated, so as to ensure the objectivity of the evaluation. It is clear from Table 2 that the correlation between the adjacent pixels is very small (or practically zero) in the shuffled images produced by the proposed and total shuffling algorithms. Unfortunately, the other four methods, which perform well on an ordinary image, may not be suitable for practical medical image permutation. This is due to the extremely unbalanced distribution of pixel values of a medical image, i.e., the pixels values are concentratedly distributed in a few small ranges.

**Table 2:** Correlation coefficients of the proposed and comparable methods for different test images.

Test image	Direction	Plain image	Proposed method	Cat map	Baker map	Standard map	Total shuffling	Chaotic sequence sorting
Head CT	Horizontal	0.976088	−0.003013	0.087212	0.171150	0.116150	−0.005588	0.170638
	Vertical	0.972538	−0.004488	0.056863	0.168963	0.371738	0.001025	0.124013
	Diagonal	0.953250	0.000650	0.070238	0.003675	0.116850	−0.000275	−0.021613
	**Average**	0.967292	−***0.002284***	0.071437	0.114596	0.201579	−***0.001613***	0.147326
Paranasal sinus CT	Horizontal	0.990000	−0.004450	0.16625	0.250925	0.113225	0.001838	0.249475
	Vertical	0.984825	−0.007245	0.135938	0.317025	0.463425	0.011628	0.175938
	Diagonal	0.976738	−0.001704	−0.041675	0.113225	0.113338	0.004275	−0.000707
	**Average**	0.983854	−***0.004466***	0.086838	0.227058	0.229996	***0.005914***	0.141569
Chest X-ray	Horizontal	0.990325	−0.000796	0.273513	0.2310625	0.190825	−0.006475	0.396925
	Vertical	0.994150	−0.004575	0.092500	0.434775	0.536525	0.003825	0.17945
	Diagonal	0.985225	−0.005708	−0.065288	0.0423125	0.1943625	−0.002975	−0.017803
	**Average**	0.989900	−***0.003693***	0.100242	0.236050	0.307238	−***0.001875***	0.186191
Waist MR	Horizontal	0.984813	−0.003450	0.320775	0.075313	0.067188	−0.0090125	0.506975
	Vertical	0.961813	−0.004025	0.144025	0.404713	0.314400	−0.007031	−0.001950
	Diagonal	0.949988	0.001763	0.078138	0.061575	0.071625	−0.000063	−0.041338
	**Average**	0.965538	−***0.001904***	0.180979	0.180534	0.151071	−***0.005369***	0.154562
Abdomen CT	Horizontal	0.956663	0.010713	0.165188	0.207663	0.130463	−0.008600	0.1561875
	Vertical	0.978300	0.011729	0.108988	0.141350	0.361513	−0.004088	0.2090625
	Diagonal	0.940175	−0.004260	−0.122938	0.053488	0.143300	−0.002250	−0.009100
	**Average**	0.958379	***0.006061***	0.050413	0.134167	0.211759	−***0.004979***	0.118717

To evaluate the computational efficiency, test images of different size are shuffled by each method ten times, and the average execution times can be found in Table 3. All the algorithms have been implemented using Code::Blocks and the tests have been done on a personal computer with an Intel Core i5-3470 CPU and 2 GB RAM. The data show that the proposed method runs only slower than the chaotic sequence sorting algorithm, whose effectiveness, however, is not satisfactory. The total shuffling algorithm, on the contrary, can produce images with desired shuffling effect but suffers from poor efficiency. Thus, it can be conclude from above analysis that the proposed permutation method provides the best trade-off between effectiveness and efficiency.

**Table 3:** Execution time of the proposed and comparable permutation methods.

Image size	Average permutation time (*ms*)					
	Cat map	Baker map	Standard map	Chaotic sequence sorting	Total shuffling	Proposed method
256×256	2.0	7.9	20.1	2.3	183.4	2.3
512×512	8.2	30.9	80.8	5.8	1492.3	8.6
1024×1024	46.3	120.5	303.7	22.0	13086.8	35.5
2048×2048	185.6	488.5	1169.1	88.1	114125.6	162.3

## Security Analysis

A good cryptosystem should be robust against all kinds of known attacks, such as brute-force attack, cipher-text only attack, differential attack, and statistical attacks. In this section, thorough security analysis has been carried out to demonstrate the high security of the proposed scheme.

### A. Key space analysis

The key space is the total number of different keys that can be used in the encryption/decryption procedure. For an effective cryptosystem, the key space should be large enough to make brute-force attack infeasible. As mentioned above, the key of the proposed cryptosystem is composed of three initial state values (*x*_0_, *y*_0_, *z*_0_)∈*R* of the Chen's system. The three variables are independent of each other, and therefore the key space of the proposed medical image cryptosystem is
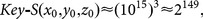
(14)which is large enough to make brute-force attack infeasible.

### B. Statistical analysis

It is well known that many ciphers have been successfully analyzed with the help of statistical analysis and several statistical attacks have been devised on them. To prove the robustness of the proposed scheme, we have performed statistical analysis by calculating the histogram, the information entropy, and the correlation of two adjacent pixels.

#### 1) Histogram

**Figure 4:**
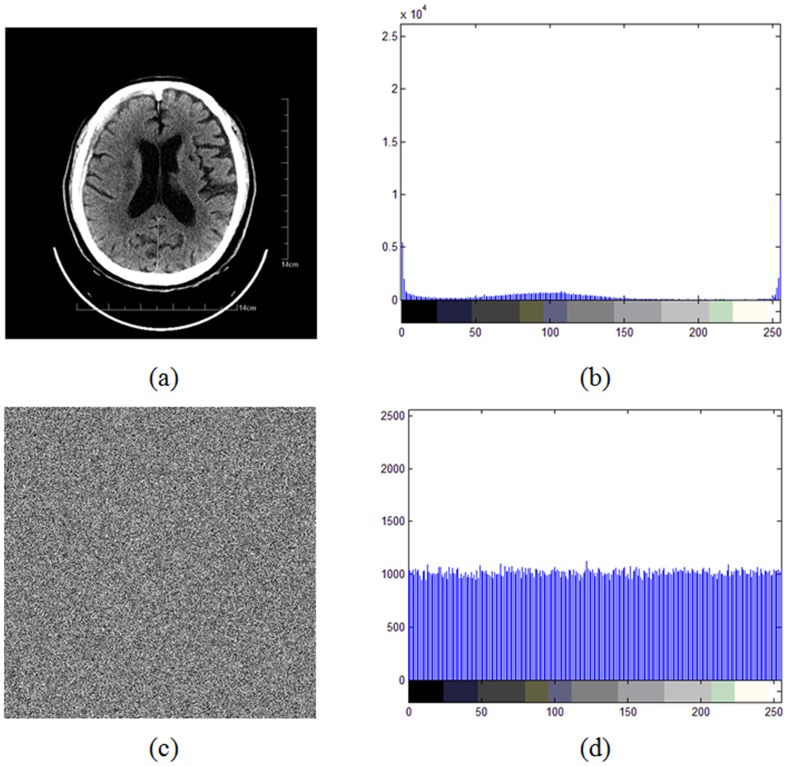
Histogram analysis: (a) plain image. (b) histogram of plain image. (c) cipher image. (d) histogram of cipher image.

The frequency distribution of cipher pixel is of much importance to an image cryptosystem. It should hide the redundancy of plain image and should not leak any information on the relationship between plain image and cipher image. An image histogram is a graphical representation of the number of pixels in an image as a function of their intensity. The histograms of the test image (Fig. 4(a)) and its ciphered image (Fig. 4(c)) produced by the proposed scheme are shown in Figs. 4(b), (d), respectively. It's clear from Fig. 4(d) that the histograms of the cipher image are fairly uniform and significantly different from that of the plain image and hence does not provide any clue to employ statistical analysis.

#### 2) Correlation of adjacent pixels

**Figure 5:**
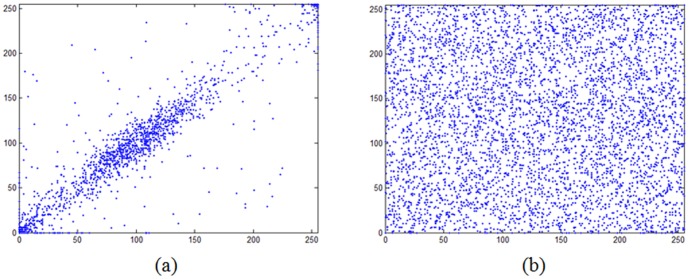
The visual testing of correlation of diagonally adjacent pixels: (a) correlation of diagonally adjacent pixels in the plain image. (b) correlation of diagonally adjacent pixels in the cipher image.

As is known, pixels in an ordinary image are usually highly correlated with their adjacent pixels either in horizontal, vertical or diagonal direction [42]. However, an efficient image cryptosystem should procedure the cipher image with sufficiently low correlation in the adjacent pixels. Besides the qualitative method employed in Sec. 3, the correlation of adjacent pixels can also be visually tested, which is carried out by plot the distribution of the adjacent pixels by using each pair as the values of the *x*-coordinate and *y*-coordinate. Figs. 5(a) and (b) show the correlation distribution of two horizontally adjacent pixels of the test image (Fig. 3(a)) and its ciphered image produced by the proposed scheme, respectively. Similar results can be obtained for horizontally and diagonally adjacent pixels. As can be seen from Fig. 5(a), most points are clustered around the main diagonal, whereas those in Fig. 5(b) are fairly evenly distributed. The simulation results indicate that the strong correlation between adjacent pixels in the plain image has been effectively eliminated in the cipher image.

#### 3) Information entropy

In information theory, entropy is the most significant feature of disorder, or more precisely unpredictability. To calculate the entropy *H*(*s*) of a source *s*, we have:
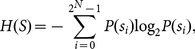
(15)where *N* is the number of bits to represent a symbol *s_i_*∈*s* and *P*(*s_i_*) represents the probability of symbol *s_i_* so that the entropy is expressed in bits. For a truly random source emitting 2*^N^* symbols, the entropy is *H*(*s*) = *N*. Therefore, for a ciphered image with 256 gray levels, the entropy should ideally be *H*(*s*) = 8. If the output of a cipher emits symbols with entropy less than 8, there exists certain degree of predictability, which threatens its security.

The information entropy of the five test images and their corresponding cipher images produced by the proposed scheme are calculated, and the results are listed in Table 4. As can been seen from Table 4, the entropy of all the output cipher images are very close to the theoretical value of 8. This means that information leakage in the encryption process is negligible and the cryptosystem is secure against entropy analysis.

**Table 4:** Results of information entropy analysis.

Test image	Head CT	Paranasal sinus CT	Chest X-ray	Waist MR	Abdomen CT
Plain	3.522034	3.328586	7.564915	6.120326	2.595700
Cipher	7.999293	7.999256	7.999297	7.999244	7.999314

#### 4) The randomness of the keystream

As is known, keystreams for cryptographic applications must be generated in a random fashion and the randomness of a keystream greatly affects the security of the cryptosystem. In order to testify the randomness of the keystream employed in our cryptosystem, a statistic test suite designed by NIST (National Institute of Standards and Technology) [43] is applied. The test suite is a statistical package consisting totally of 16 tests, evaluating three major aspects of randomness of a binary sequence, namely,

(1) Random walk: the frequency (monobit) test, frequency test within a block, the cumulative sums (cusums) test, the random excursions test, and the random excursions variant test.

(2) Pattern checking: the runs test, tests for the longest-run-of-ones in a block, the non-overlapping template matching test, the overlapping template matching test, Maurer's “universal statistical” test, the serial test, and the approximate entropy test.

(3) Complexity and compression: the binary matrix rank test, the discrete Fourier transform (spectral) test, and the linear complexity test.

The test is carried out as follows. For each statistical test, a set of *P-values* (corresponding to the set of sequences) is produced. A sequence passes a statistical test whenever the *P-value* ≥*α* and fails otherwise, where *α*∈(0.001, 0.01] is the significance level. For each statistical test, compute the proportion of sequences that pass. For example, if 1000 binary sequences were tested, *α* = 0.01, and 997 binary sequences had *P-values* ≥0.01, then the proportion is 997/1000 = 99.70%.

The range of acceptable proportions is determined using the confidence interval defined as
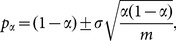
(16)where *σ* is the number of standard deviations and *m* is the sample size. In our experiments, 100 sequences (*m* = 100), each with 1,000,000-bit long, are generated with randomly selected diffusion keys. Together with the chosen standard parameters, *α* = 0.01 and *σ* = 3, we have 96.02%≤*P_α_*≤100.00%. If the proportion falls outside of this interval, then there is evidence that the data is non-random. The test results are shown in Table 5, from which it can be seen that all the 16 tests are passed, and hence the keystream generated by the proposed scheme are suitable for cryptographic usage.

**Table 5:** Passing rate of the keystream generated by the proposed scheme.

Test items	Passing rate
Frequency	97.00%
Block Frequency	98.00%
Cumulative Sums forward sum	98.00%
Cumulative Sums backward sum	97.67%
Runs	98.00%
Longest Run	98.00%
Rank	97.00%
FFT	97.00%
NonOverlapping Template	97.76%
Overlapping Template	98.00%
Universal	97.00%
Approximate Entropy	98.00%
Random Excursions	99.32%
Random Excursions Variant	99.09%
Serial	97.00%
Linear Complexity	97.00%

### C. Key sensitivity analysis

This test is intended to emphasize the diffusion property of the proposed cryptosystem under consideration with respect to small changes in keys. This is important because otherwise an intruder might reconstruct parts of the plain image from the observed cipher image by a partly correct guess of the key used for encryption. The key sensitivity of an image cryptosystem can be observed in two ways: (1) completely different cipher images should be produced when slightly different keys are used to encrypt the same plain image; (2) no data can be recovered from cipher image even though there is only a minor difference between the encryption and decryption keys.

**Figure 6:**
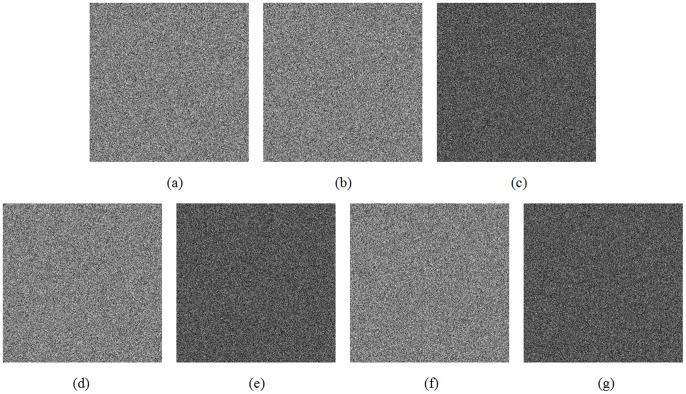
Results of key sensitivity test 1.

To evaluate the key sensitivity of the first case, the test image (Fig. 3(a)) is encrypted using four slightly different test keys, respectively, as listed in Table 6. The corresponding cipher images are shown in Figs. 6(a), (b), (d) and (f), respectively. The differential images between (a) and (b), (a) and (d), and (a) and (f) are shown in (c), (e) and (g) of Fig. 6, respectively. Moreover, the differences between any two cipher images are computed and also given in Table 6. As can be seen from Fig. 6 and Table 6, the four cipher images show no similarities at all and there is no significant correlation that could be observed from the differential images.

**Table 6:** Differences between cipher images produced by slightly different keys.

Figure	Test key			Differences			
	*x*_0_	*y*_0_	*z*_0_	8(a)	8(b)	8(d)	8(f)
8(a)	5.82249164527227	8.69941032358007	−2.64779026475630	——	99.60%	99.61%	99.61%
8(b)	***5.822491645***27228	8.69941032358007	−2.64779026475630	99.60%	——	99.61%	99.63%
8(d)	5.82249164527227	***8.699410323***58008	−2.64779026475630	99.61%	99.61%	——	99.63%
8(f)	5.82249164527227	8.69941032358007	−***2.647790264***75629	99.61%	99.63%	99.63%	——

**Figure 7:**
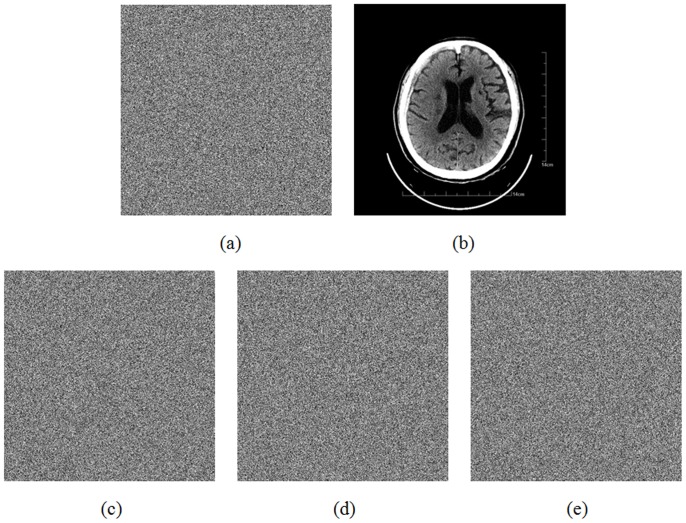
Results of key sensitivity test 2.

To evaluate the key sensitivity of the second case, the test image (Fig. 3(a)) is firstly encrypted using the test key (*x*_0_ = 8.79013904597178, *y*_0_ = −9.88911616079589, *z*_0_ = 5.22375356944771) and the resultant cipher image is shown in Fig. 7(a). Then the ciphered image is tried to be decrypted using four decryption keys: (i) (*x*_0_ = 8.79013904597178, *y*_0_ = −9.88911616079589, *z*_0_ = 5.22375356944771), (ii) (*x*_0_ = ***8.79013904597177***, *y*_0_ = −9.88911616079589, *z*_0_ = 5.22375356944771), (iii) (*x*_0_ = 8.79013904597178, *y*_0_ = −***9.88911616079590***, *z*_0_ = 5.22375356944771) and (iv) (*x*_0_ = 8.79013904597178, *y*_0_ = −9.88911616079589, *z*_0_ = ***5.22375356944770***). The resultant decrypted images are shown in Figs. 7(b), (c), (d) and (e), respectively. The differences between the wrong deciphered images (c), (d) and (e) to the plain image are all 99.61%.

The results of above two tests indicate that the proposed scheme is highly sensitive to the key. Instead any attempt to decrypt with a wrong key is in fact another encryption operation.

### D. Differential analysis

To implement differential attack, an opponent usually makes a slight change, usually one pixel, in the plain image and ciphers the two images using the same secret key. If some meaningful relationship between the plain image and cipher image can be found by comparing the two cipher images, the secret key may be determined with the help of some other analysis methods. This kind of cryptanalysis may become inefficient and practically useless if one minor change in the plain image can be effectively diffused to the whole ciphered image. To test the influence of one pixel change on the whole image, two common measures *NPCR* (number of pixel change rate) and *UACI* (unified average changing intensity) are used.

The *NPCR* is used to measure the percentage of different pixel numbers between two images. Let *P*_1_(*i*, *j*) and *P*_2_(*i*, *j*) be the (*i*, *j*)th pixel of two images *P*_1_ and *P*_2_, respectively, the *NPCR* can be defined as:



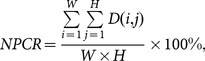
(17)where *D*(*i*, *j*) is defined as



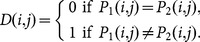
(18)The *NPCR* value for two random images, which is an expected estimate for a good image cryptosystem, is given by



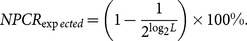
(19)


For instance, the expected *NPCR* for two random images with 256 gray levels is 99.609%.

The second criterion, *UACI* is used to measure the average intensity of differences between the two images. It is defined as



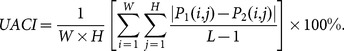
(20)


The *UACI* value for two random images is given by



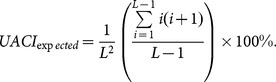
(21)


For a 256 gray levels image, the expected *UACI* value is 33.464%.

In our simulations, we assume a worst case that two plain images have only 1-bit difference at the lower right corner pixel. The *NPCR* and *UACI* values of different test images are calculated and listed in Table 7. Each image pair is encrypted under the same key and two rounds of encryption is adopted. It is clear that the *NPCR* and *UACI* values are very close to the expected values, and hence the proposed scheme has a strong ability against differential attack.

**Table 7:** *UACI* and *NPCR* results (in %) using different plain images.

Test image	Head CT	Paranasal sinus CT	Chest X-ray	Waist MR	Abdomen CT
*NPCR*	99.600	99.639	99.604	99.607	99.618
*UACI*	33.482	33.514	33.531	33.388	33.484

### E. Speed performance

Table 8 shows the time required for encrypting a 512×512 image with 256 grey levels by using the proposed and some typical block and chaos-based approaches. The number of permutation/diffusion rounds indicate the minimum number of iterations required to achieve a satisfactory diffusion effect, i.e., *NPCR*>0.996 and *UACI*>0.334. All the tests have been done on the same hardware mentioned in Sec. 3. As the operation mechanism of the chaos-based encryption algorithms is quite different from that of block algorithms, the comparison of iteration times is made only between chaos-based approaches. It's clear from Table 8 that the proposed scheme has the highest operating efficiency. The speedup is mainly due to the following two improvements. (1) Generally, a chaos-based image cipher utilizes two different chaotic maps/systems to generate the permutation table and diffusion keystream, respectively. To accomplish a cipher, both processes require a tremendous number of iterations of a chaotic map/system. While in our scheme, a single chaotic system is employed for both permutation and diffusion. (2) As mentioned above, in conventional schemes, the order of the diffusion operation in each round of iteration is fixed, i.e., from left to right and top to bottom. While in our scheme, the order is changed in every other round. With such a mechanism, less number of rounds is required to achieve a satisfactory diffusion effect. Both these strategies suggest an effective way of reducing the computational complexity of an image cryptosystem. With such a speed, the proposed scheme is particularly suitable for real-time teleradiology applications which facilities emergency remote triage and diagnosis.

**Table 8:** Comparison between the performance and security of the proposed and some typical block and chaos-based ciphers.

Approaches	Total cites	Number of chaotic systems employed	Permutation rounds	Diffusion rounds	Known/chosen- plaintext attack	Encryption time (*ms*)
AES	N/A	N/A	N/A	N/A	Robust (CBC)	302.6
Fridrich (1998) [6]	734	2	4	4	Weak	163.2
Chen et al (2004) [8]	1024	2	4	4	Weak	156.7
Patidar (2009) [20]	132	2	2	2	Weak	146.9
Wang et al (2009) [22]	83	2	2	2	Robust	48.1
Zhu et al (2011) [28]	91	2	2	2	Weak	83.3
Our scheme	N/A	1	2	2	Robust	44.1

## Conclusions

This paper has suggested a novel chaos-based image cipher for medical image protection. The new scheme utilizes a single 3D chaotic system, Chen's chaotic system, for both permutation and diffusion. To address the security and efficiency problems encountered by many existing permutation-substitution type image ciphers, we introduced a novel permutation mechanism, which shuffles each pixel in the plain image by swapping it with another pixel chosen by two of the three state variables of Chen's chaotic system. The remaining variable is used for quantification of pseudorandom keystream for diffusion. Results of permutation performance analysis have shown that the new permutation method outperforms existing methods with respect to either effectiveness or efficiency. In addition, the selection of state variables is controlled by the plain pixel. As a result, the quantified keystream is related to both the key and the plain image, which enhances the security against known/chosen-plaintext attack. Extensive security analysis has been performed on the proposed scheme, including the most important ones like key space analysis, key sensitivity analysis, differential analysis and various statistical analyses, which has demonstrated the satisfactory security of the proposed scheme. The running speed of the proposed scheme is tested and compared with that of some typical block and chaos-based approaches. The results have shown the superior performance of the proposed scheme. In conclusion, the proposed medical image protection scheme is particularly suitable for real-time telemedicine applications.
